# Lightweight Techniques to Improve Generalization and Robustness of U-Net Based Networks for Pulmonary Lobe Segmentation

**DOI:** 10.3390/bioengineering11010021

**Published:** 2023-12-25

**Authors:** Armin A. Dadras, Achref Jaziri, Eric Frodl, Thomas J. Vogl, Julia Dietz, Andreas M. Bucher

**Affiliations:** 1 Division of Phoniatrics-Logopedics, Department of Otorhinolaryngology, Medical University of Vienna, Währinger Gürtel 18-20, 1090 Vienna, Austria; 2 Center for Cognition and Computation, Goethe University Frankfurt, Robert Meyer Str. 10-12, 60323 Frankfurt am Main, Germany; 3 Institute for Diagnostic and Interventional Radiology, University Hospital, Goethe University Frankfurt, Theodor-Stern-Kai 7, 60590 Frankfurt, Germanyjulia.dietz@em.uni-frankfurt.de (J.D.); 4 Department of Medicine, Medical Clinic 1, University Hospital, Goethe University Frankfurt, Theodor-Stern-Kai 7, 60590 Frankfurt, Germany

**Keywords:** artificial intelligence, lung thorax, CT, segmentation, deep learning, computer vision, self-supervised learning, attention

## Abstract

Lung lobe segmentation in chest CT is relevant to a wide range of clinical applications. However, existing segmentation pipelines often exhibit vulnerabilities and performance degradations when applied to external datasets. This is usually attributed to the size of the available dataset or model. We show that it is possible to enhance generalizability without huge resources by carefully curating the dataset and combining machine learning with medical expertise. Multiple machine learning techniques (self-supervision (SSL), attention (A), and data augmentation (DA)) are used to train a fast and fully-automated lung lobe segmentation model based on 2D U-Net. Our study involved evaluating these techniques on a diverse dataset collected under the RACOON project, encompassing 100 CT chest scans from patients with bacterial, viral, or SARS-CoV2 infections. We compare our model to a baseline U-Net trained on the same dataset. Our approach significantly improved segmentation accuracy (Dice score of 92.8% vs. 82.3%, *p* < 0.001). Moreover, our model achieved state-of-the-art performance (Dice score of 92.8% vs. 90.8% for the literature’s state-of-the-art, *p* = 0.102) with reduced training examples (69 vs. 231 CT Scans). Among the techniques, data augmentation with expert knowledge displayed the most significant impact, enhancing the Dice score by +0.056. Notably, these enhancements are not limited to lobe segmentation but can be seamlessly integrated into various medical imaging segmentation tasks, demonstrating their versatility and potential for broader applications.

## 1. Introduction

Segmenting lesions is a mandatory step for quantifying medical imaging data and enables the development of quantitative imaging biomarkers [1]. Quantification allows extending the diagnostic tools in research and clinical settings beyond subjective image interpretation. A range of measurement and classification tasks can be based on segmentation masks, leading to a much more comprehensive characterization of patient collectives using imaging biomarkers [2]. This also applies to lung anatomy. It contains fundamental information that can be leveraged by a wide range of clinical applications. Chest CT is the primary diagnostic imaging modality for the classification of most pulmonary disease entities. Information about localization, volume, or shape of each lobe structure is a prerequisite to providing a precise diagnosis of pulmonary diseases and planning treatments. The lung is typically composed of five lobes, separated by the lobar fissures with some anatomic variety. Fissures can be visualized as thin structures on high-resolution CTs. However, this visualization is often incomplete or even missing due to spatial resolution or artifacts. Additionally, even under ideal imaging conditions, underlying thoracic pathology or previous surgical interventions can substantially change morphology as well as the location of lobe structures. These include pleural effusions, pulmonary consolidations, malignant lesions, and incomplete or complete lung lobe resections.

Unfortunately, segmenting huge amounts of data manually is very labor-intensive and not feasible in most cases. Automating the segmentation step is, therefore, crucial for further processing. Earlier approaches for lobe segmentation incorporated image processing and leveraged domain information to solve the task. Anatomical relations between lobes and nearby airways, vessels, and the lung borders were exploited to account for incomplete fissures and damaged lung [3,4,5,6]. For instance, ref. [7] proposed a method to model sheet-like, tubular, and blob-like structures by the eigenvalues of the Hessian matrix. This method worked well in normal cases but was sensitive to imaging parameters such as CT scan protocols/parameters, image noise, and the presence of underlying lung diseases (e.g., chronic obstructive pulmonary disease). Other approaches for automatic lung segmentation include thresholding, surface fitting [8], water-shedding [9], and graph searching with shape constraints [10]. These model-driven approaches fail to generalize and are sensitive to small changes in the quality of input images, which hinders their application in a clinical setting.

Data-driven designs have seen a resurgence in recent years, thanks in particular to recent advances in computational optimization methods. Architectures like deep convolutional neural networks (CNNs) have the advantage of being more scalable. These approaches build on the assumption that invariances can be learned with enough data. Coupled with extensive testing based on rigorous engineering principles, they provide state-of-the-art performance for many tasks.

Many works have successfully adopted 3D or 2D CNNs in a lobe segmentation framework. These approaches yielded good results without the need for domain expertise and modeling. Various ideas were explored to improve performance such as multitasking [11,12], dense networks [13], leveraging global geometric features as additional inputs [14], cascaded networks for global and local features [15], holistically nested network [16,17] and advanced loss functions to tackle class imbalance issue [18]. However, these techniques offer only minor improvements, mainly due to limitations in the datasets used for training, which have been shown to be the main source of errors [19]. For example, Park et al. performed pulmonary lobe segmentation using a 3D convolutional neural network to develop a robust algorithm without lobar fissure detection and outperformed image processing–based segmentation in terms of accuracy and execution time [20]. Validation using internal and external datasets demonstrated that their method could be applied to clinical radiology. However, their study only considered mild-to-moderate COPD patients. Thus, the presented method fails when the lung disease becomes severe, and the lung structures change considerably.

Automatic CT segmentation pipelines, especially on the lung, are vulnerable to many types of possible perturbations. Healthy and normal lungs can be segmented accurately, but CT segmentation pipelines cannot handle cases that deviate from the norm. Anatomical variabilities concerning the shape, size, or even number of lobes, as well as pathologies, such as fibrosis or consolidations can result in an insufficient delineation of interlobar fissures [21]. During the data acquisition process, various factors, such as the type and manufacturer of the scanning device or the contrast materials administered to the patient, impact the appearance of the lungs. For a safe deployment in clinical settings, an automatic segmentation pipeline should exhibit robustness to these perturbations and should work on different data-capturing protocols.

In this study, we follow on from the work of Hofmanninger et al. [19] to show that instead of heavy engineering and huge datasets, the focus should be on data quality and exchange between computer scientists and domain experts. We curated a diverse dataset, an in-house dataset that covers a wide range of challenging cases. These are then carefully segmented by medical experts to obtain the ground truth. Subsequently, we present a neural network system that combines novel self-supervised pre-training (SSL), attention gates (A), and data augmentation with expert knowledge (DA) to get the most out of our data. We analyze the three techniques (SSL, DA, A) to train a fast and fully-automated lung lobe segmentation model based on U-Net and contrast the performance on publicly available datasets. Our results highlight the soundness of our proposed design choices, outperforming other baselines.

## 2. Materials and Methods

**Figure 1:**
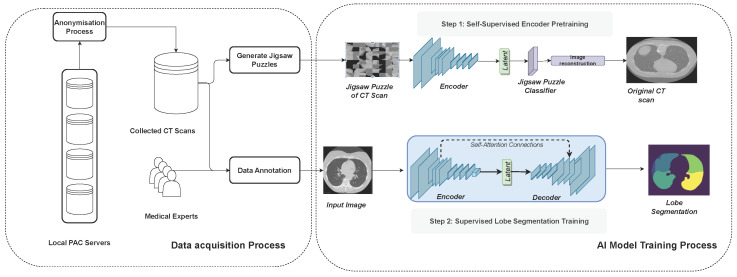
Diagram to illustrate the methodology adopted in this work. Data collection involves meticulous acquisition from local PAC servers, guided by medical experts’ insights. The selected data undergoes multiple rounds of annotation by radiology experts. Subsequently, the CT scan data are utilized for self-supervised training, enhancing the encoder network’s representation extraction abilities. Finally, the pre-trained encoder is used in the encoder-decoder architecture for end-to-end training.

In this section, we outline the process undertaken for data collection, as well as the design and implementation of our deep learning model. Figure 1 illustrates the full methodology followed in this work.

### 2.1. Data Acquisition

Following the findings of Hofmanninger et al. [19], we designed a dataset that covers a wide range of variations for lobe segmentation (Table 1). We selected 100 CT scans from the local PACs, resembling different degrees of disease severity, distributed among three categories of pneumonia. These were COVID-19 (50%), bacterial (25%), or viral (25%). Parameters, such as slice thickness and the convolution kernel, which are used to create reconstructions from raw CT data, vary from case to case and may influence the accuracy. Therefore, we also picked cases of up to 5 mm thickness per slice and different kernels. Contrast media are commonly given to the patient to improve the contrast resolution and, therefore, change the visual appearance of the image. We picked a portion of our data such that this parameter is covered in every class of disease. The image series were subsequently manually segmented by six medical experts and underwent iterative control rounds by a senior radiologist with 8 years of experience in reading chest CTs. In order to prevent quality issues in experiments [22] we followed an established reporting guideline to keep track of the imaging setting [1].

For the evaluation of our model, we rely on public datasets (LUNA: [23], IEE: [24]). These were excluded from the training procedure to investigate the generalization properties of our model.

### 2.2. Data Pre-Processing

In the preprocessing phase, we applied several essential transformations to optimize the input data for subsequent analysis. The initial step involved resampling the images to achieve a uniform spacing of 2 mm between slices. For this purpose, we employed the basis spline function [25] from the SimpleITK (SITK) library, utilizing its interpolation capabilities to ensure the preservation of relevant details during the resampling process. Following resampling, a crucial normalization step was undertaken, scaling the pixel intensity values to a standardized range between 0 and 1. This normalization facilitates consistent data representation across the dataset, contributing to the stability and effectiveness of subsequent computational processes. Moreover, to streamline the computational load and enhance computational efficiency, each CT slice was downsized to a resolution of 256×256 pixels. This downsizing not only expedites computational analyses but also ensures that the model effectively captures salient features while mitigating unnecessary computational burdens. Together, these preprocessing steps lay the foundation for robust and standardized input data, optimizing the subsequent stages of our analysis pipeline.

### 2.3. Models

For many medical segmentation tasks, deep learning has led to a leap in performance. The U-Net Architecture [26] in particular has shown good results for segmentation of biomedical data due to its ability to preserve information along different levels of abstraction using to skip connections (Table 2).

We apply a state-of-the-art version of it to our lobe segmentation task as a baseline for our analysis. In every down- and up-sampling step, we apply two convolutions, together with BatchNorm and ReLu activations. Our output results in 6 channels, which encode the 5 lobes and a background class. We optimize the U-net network for each of the tasks by applying the multi-class Dice loss.

Although the appearance and textures of CT scans are dependent on the approximations of the chosen CT scanning device, shapes tend to be invariant and more generalizable across different data-capturing protocols. We hypothesize that encouraging the deep learning model to learn geometric contextual features can help address the issue of domain shift and improve the lung segmentation capabilities of our model on out-of-distribution data.

To this end, we consider three modeling choices to improve the robustness of our models.

- Self-Attention
- Self-Supervised pre-training
- Expert Guided Data Augmentation

All our code is written in Python 3.7 using PyTorch 1.8 and trained on a local computer using Nvdia GA102 GPUs.

### 2.4. Self-Supervised Pre-Training

Self-supervised learning (SSL) provides effective representations for downstream tasks without requiring human labeling. Ref. [27] improve classification accuracy by employing a self-supervised auxiliary learning task in which they predict image rotations (Figure 1). The experimental results show that this type of SSL increases the robustness against different kinds of perturbations, ranging from adversarial attacks to motion blur and Gaussian noise. Additionally, self-supervision greatly benefits from out-of-distribution detection on difficult, near-distribution outliers. The work of [28] further demonstrated robust improvements in the context of multi-organ segmentation.

Therefore, we considered self-supervised pre-training to improve the robustness of our lobe segmentation approach.

As a self-supervision task, we choose to solve jigsaw puzzles, which requires no manual labeling. Jigsaw puzzle training is a technique used to enhance a model’s ability to understand spatial relationships and context within images. The input image is broken into smaller pieces, and the goal is to reconstruct the original image by rearranging these pieces. The input images are divided into smaller patches or tiles. These patches are shuffled or rearranged to create a jigsaw puzzle. Our model is then trained to predict the correct arrangement of these shuffled patches to reconstruct the original image. Instead of predicting traditional labels or categories, the model learns to arrange these patches spatially. The loss function, the cross-entropy loss, quantifies the difference between the predicted permutation of patches and the actual permutation. These are represented as a distance metric between the predicted permutation and the ground truth permutation. Table 3 details the jigsaw puzzle classification module. After training the model to predict the permutation of patches, the encoder is fine-tuned for lobe segmentation tasks with labeled data.

### 2.5. Attention U-Net

Attention mechanisms play a pivotal role in enhancing image classification performance by enabling class-specific pooling, thereby fostering greater accuracy and robustness. The utility of attention maps lies in their ability to amplify pertinent regions within an image, showcasing superior generalizability across multiple benchmark datasets [29]. A noteworthy contribution to the field is presented by [30], introducing a novel attention gate (AG) model specifically tailored for medical imaging segmentation. This AG model autonomously learns to focus on target structures, seamlessly integrated into a U-Net framework. The U-Net model, trained in conjunction with attention gates, inherently acquires the capability to suppress irrelevant regions within an input image while accentuating salient features critical for the specific segmentation task. This unique attribute eliminates the need for explicit algorithms dedicated to lung localization, enabling the end-to-end learning of pulmonary lobe structures. Consequently, the incorporation of attention gates into the U-Net architecture not only enhances model sensitivity and accuracy in identifying foreground pixels but also achieves this without imposing significant computation overhead. Moreover, attention gates exhibit a progressive ability to suppress feature responses in irrelevant background regions, further contributing to the model’s efficiency in focusing on diagnostically relevant areas during medical image segmentation.

### 2.6. Data Augmentation

Data augmentation, a commonly used technique in enhancing generalizability and accuracy, proves especially invaluable in scenarios where data availability is limited, a common challenge in medical imaging. Given the substantial data requirements of deep learning models and the inherent difficulty in obtaining sizable medical datasets, data augmentation emerges as a crucial strategy. This technique involves applying a diverse range of transformations to existing data, thereby introducing variations that mimic real-world scenarios encountered in clinical scans, such as tissue deformations or scanning artifacts. The rationale behind these transformations is to simulate plausible variations, aligning with the intricacies observed in actual medical imaging. In our experimental approach, we collaborated closely with medical professionals, allowing us to qualitatively define parameters within expert-defined limits. This collaborative effort ensures that the resulting augmentations maintain clinical plausibility, aligning with the nuanced variations present in authentic medical scans. To implement these transformations, we utilized the *Albumentation* library [31], a versatile tool that facilitates the application of modifications to medical images, contributing to the robustness and realism of our augmented dataset.

**Figure 2:**
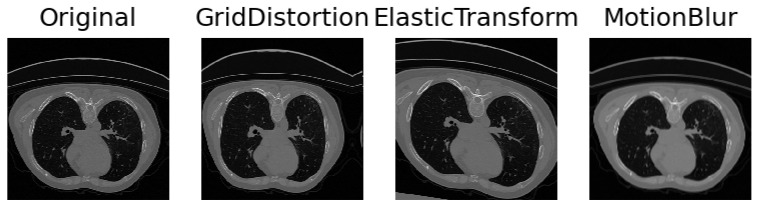
Figure illustrating the data transformations used in the training phase to improve the capabilities of our proposed model to perform on different anatomies or acquisition settings. From left to right: Original image, Grid distortion, Elastic transformation, Motion blur. The visible effect of these transformations varies due to random values during training.

Collaborating closely with medical experts, we engaged in comprehensive discussions to discern the myriad facets of variability inherent in medical imaging and intuitively selected parameters to simulate these aspects. The inherent diversity in CT scans, stemming from variations in patient anatomy and imperfect settings, necessitates the incorporation of realistic simulations. To capture anatomical variations or pathological lung anomalies, we explored geometric deformations [32], such as elastic deformation [33] and grid distortion [31]. Notably, grid distortion proved especially pertinent in mimicking anomalies arising from variations in lung geometry due to diverse anatomies or pathologically expanded lungs. In addition to geometric deformations, we introduced Gaussian noise and blur to replicate movements and inhalation dynamics during the scanning procedure. Recognizing that scanner and reconstruction errors contribute to the creation of noisy images, we strategically simulated these imperfections. Further, adjustments to contrast and brightness were implemented to approximate the diverse settings across different scanner types and reconstruction parameters. The results can be seen in Figure 2. A meticulous observation of the resulting images guided us in identifying the settings that yielded the most realistic simulations, ensuring that our augmented dataset encapsulates the intricate variability present in authentic medical scans.

The meaning of the parameters in Table 4 are described in the referred papers or the albumentation documentation (see https://albumentations.ai/docs/api_reference/augmentations/transforms/, accessed on 15 November 2022). They may vary depending on the preprocessing and normalization steps performed.

### 2.7. Training Specifications

The models were trained and validated using the Pytorch framework. To initialize our models, we used Xavier initialization [34]. In the Xavier procedure, the weights are initialized so that the variance of the activation layers is the same across every layer. This helps to prevent the gradient from exploding or vanishing. Additionally, we used Adam as a method for stochastic optimization with an initial learning rate of $10^{-4}$ and weight decay of $10^{-5}$. The learning rate was multiplied by 0.96 after 2 epochs. As a stopping criterion, we trained all models for 50 epochs and saved the best-performing model on the validation set. A mini-batch of 16 images was used. The training set was composed of around 9800 CT slices in total, stemming from 69 3D CT scans. For the cost function, we employed a multi-class Dice as suggested in [12]. This loss properly handles the class imbalance problem prevalent in lung lobe segmentation: lung lobes have different sizes, and background regions can constitute a large part of the image. For each lobe class k, we computed a Dice score $D_{k}$ as such:

(1) $$D_{k}=\frac{2\cdot \sum_{i}^{N}p_{i}^{k}g_{i}^{k}}{\sum_{i}^{N}{\left(p_{i}^{k}\right)}^{2}+\sum_{i}^{N}{\left(g_{i}^{k}\right)}^{2}}$$

where *N* is the number of pixels, $p^{k}$ the binary segmentation map for class *k* and $g^{k}$ the ground truth for class *k*. The final loss value is the average of Dice scores for all the classes.

### 2.8. Evaluation Measure

The performance of our model was rigorously assessed using the widely adopted Dice coefficient for semantic segmentation, encompassing a comprehensive evaluation of 218 instances. The Dice coefficient serves as a robust metric, quantifying the similarity between the predicted segmentation map (*P*) and the corresponding ground truth (*G*). This metric provides valuable insights into the accuracy and efficacy of our model’s segmentation predictions, offering a quantitative measure of the overlap between the predicted and actual segmentations. A higher Dice coefficient signifies greater concordance, indicating the model’s proficiency in capturing the nuances of the target structures within the images. The meticulous evaluation across different datasets ensures a comprehensive understanding of the model’s performance across a diverse range of scenarios, reinforcing the reliability and versatility of our segmentation approach. Dice is defined as:

(2) $$\begin{array}{c} DSC\left(P,G\right)=\frac{2\cdot TP}{2\cdot TP+FP+FN} \end{array}$$

where *TP*, *FP*, and *FN* are true positive, false positive, and false negative rates, respectively.

## 3. Results

In this section, we present the results of our study. We start by investigating the impact of the different design choices and then present our results for the full model and contrast it with the other chosen baselines.

### 3.1. Impact of Self-Supervised Pre-Training

**Figure 3:**
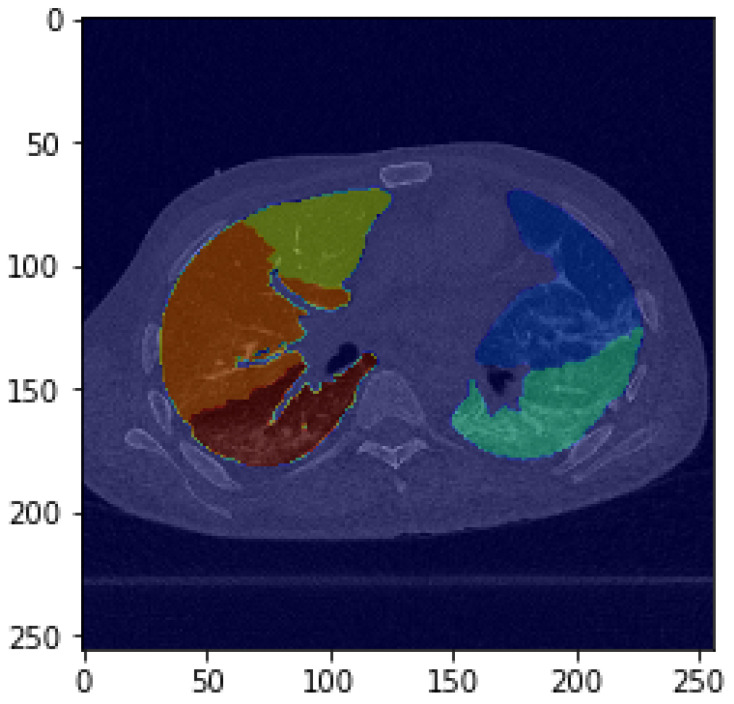
Example of a segmented lung from the evaluation set. The five lobes are delineated with different colors in this axial view.

We considered the impact of self-supervised pre-training in the context of lobe segmentation (see Figure 3 for an example). We report the performance comparison between (i) a U-Net fined-tuned on the target task after using the encoder module in learning the self-supervised task (SSL U-Net) and (ii) the baseline U-Net trained in a fully-supervised manner. Table 5 shows the average Dice score along with its standard deviation on different datasets.

Self-supervised pre-training results in a consistent improvement compared to fully-supervised training. The improvements are marginal in the case of in-distribution data. However, when we consider the external datasets, we observe a clear improvement in the performance. This indicates that there is an increased robustness and generalizability to out-of-distribution datasets. The results obtained here further confirm/support the results obtained by [27,28], where they observed that self-supervision contributes to the robustness of networks in segmentation and classification tasks.

### 3.2. Impact of Attention Gates

Table 6 compares the performance of the baseline U-Net and the Attention U-Net model. The Attention U-Net model outperforms the baseline U-Net on all test sets. The addition of Attention Gates can improve model sensitivity and accuracy without an important additional computational overhead. Since attention gates generate soft region proposals implicitly and highlight salient features useful for a specific task, the network learns to focus on target structures without additional supervision. Therefore, it is not necessary to add an external network to detect the region of interest.

### 3.3. Impact of Data Augmentations

Next, we analyzed various augmentation techniques and their impact on performance. We defined two classes of augmentations: patient-related augmentation, which slightly modifies the shape of the lungs, and scanner augmentation, which modifies the contrast and brightness of the images.

As demonstrated in Table 7, we report performance improvements for both classes of data augmentation compared to baseline. Moreover, combining both classes of data augmentations improves the performance even further. Generally, data augmentation is particularly advantageous in scenarios where insufficient training data are available, but it also helps to increase the robustness of features towards unseen data. This seems to be the case for the Luna dataset, which profits the most from these augmentations. Interestingly, the accuracy drops by three percent for the in-house dataset on which the training was performed. So, there seems to be a tradeoff in this case, which might be attributed to the introduced variance in the data that makes it harder for the model to learn specific features for the training dataset.

### 3.4. Model Comparison

Finally, we combined these design choices in a single end-to-end model. Table 8 compares our results with the state-of-the-art model by [19]. The combination of these techniques improves the segmentation performance compared to the standard U-Net. Our model achieves similar results to the module presented on external datasets by [19] even though we used only a single end-to-end neural network model, and we used fewer CT scans for training.

## 4. Discussion

We observed that data augmentation, even when the simulated scenarios are not strictly realistic, plays an essential role in extracting robust features. However, this augmentation strategy comes with a trade-off, as it leads to a slight reduction in accuracy on our in-house dataset.

To address this challenge and strike a balance between feature richness and precision, we introduced attention gates and self-supervision into our model. These additions not only mitigate the impact of data augmentations on our in-house dataset but also guide the neural network to expand its feature applicability while focusing on more precise and clinically relevant features. The synergy of these elements, data augmentation, attention gates, and self-supervision, emerges as an effective combination for our specific case, each contributing in a complementary manner to bolster overall robustness and generalizability across diverse acquisition sites.

None of the techniques used require heavy computations and apply especially well to our use case. The model generalizes to external datasets, even on hard cases. This ability can be attributed to the inclusion of visualization errors, anatomical differences, or pathologies in the training procedures. By exposing the model to a diverse range of scenarios during training, it becomes adept at handling complex cases, ensuring that its accuracy does not waver during inference. This resilience is observed consistently across different diseases, including chronic obstructive pulmonary disease (COPD) and the distinctive challenges posed by COVID-19. The model’s ability to maintain accuracy across diverse conditions underscores its reliability and applicability in real-world medical scenarios, where variations in imaging quality, anatomy, and disease manifestations are prevalent. The interdisciplinary engineering approach opens new directions toward finding novel self-supervision tasks and data augmentations specifically tailored for robustness and generalizability in medical imaging. It is important to note that the augmentation parameters that were used depend to some degree on the data on which they were performed. It is advised to manually investigate the resulting images before applying them to the training procedure. How they change with respect to acquisition parameters could be a viable question for research. Moreover, further investigations of the relationship between dataset size and training accuracy could bear potential for efficient resource allocation in research. Our experiments show that a more diverse and selected choice of scans and the interdisciplinary work of medical experts and computer scientists have led to significant improvements. It is open for discussion if this paradigm is beneficial to pure data-driven approaches in every case.

## 5. Conclusions

In this study, we have built a model tailored for lung lobe segmentation, strategically incorporating lightweight techniques gleaned from existing literature. Our findings showcase that these integrated extensions contribute significantly to enhancing both performance and robustness, all achieved without the need for additional annotation or task-specific modules. Figuring out with medical experts which challenges have to be addressed was essential to the engineering process. It made us concentrate on generating robust features, which focus more on shapes, and create data augmentations guided by generalizable findings. Remarkably, our model performed well, despite working with a relatively small dataset of 100 CTs (69 train). The strength of our approach lies in the careful curation of a diverse dataset and an exhaustive exploration of the feature space. This meticulous strategy resulted in achieving state-of-the-art accuracy, challenging the conventional notion that large datasets or computationally intensive architectures are indispensable for successful training algorithms. By exploiting the diversity inherent in our dataset, our model demonstrates a capability to excel without an overreliance on extensive data or resource-intensive architectures, emphasizing the potential efficiency of our approach in medical imaging applications.

## Figures and Tables

**Table 1:** Table highlighting the main properties of the collected data and the acquisition protocol.

Property	Value
Dataset size	100
Comorbidity	COVID-19: 50, Bacterial: 25, Viral: 25
Segmented Labels	Upper Lung Left, Lower Lung Left, Upper Lung Right, Middle Lung Right, Lower Lung Right
Region of Interest	Thorax: 88, Thorax + Abdomen: 12
Imaging Modality	CT
In-Plane Resolution	512 × 512
Slice Thickness	1 mm: 81, 2 mm: 2, 3 mm: 3, 5 mm: 14
Convolution Kernel	I70f: 30, Bl64d: 28, B60f: 12, Other: 30
Contrast Medium used	viral: 44%, bacterial: 64%, COVID-19: 24%
CTDI Medium	7.29
KVP	100 kvp: 35, 120 kvp: 54,Other (90–150): 11
Manufacturer	Siemens: 98, General Electric:2
Partition	Train/Test: 91, Validation: 9

**Table 2:** Description of the U-Net Architecture. The encoder-decoder architecture is U-shaped and consists of a contracting path to capture context and a symmetric expanding path to achieve precise localization. It combines high-level semantic information with low-level details using skip connections (+) to the expanding path.

Operation Block	Channels, Size of Filter, Padding Value	Size of Output
Input	−, −, −	(1,256,256)
Conv-BatchNorm-Relu (2×)	1, 3×3, 1	(64,128,128)
Conv-BatchNorm-Relu (2×)	64, 3×3, 1	(128,64,64)
Conv-BatchNorm-Relu (2×)	128, 3×3, 1	(256,32,32)
Conv-BatchNorm-Relu (2×)	256, 3×3, 1	(512,16,16)
Conv-BatchNorm-Relu (2×)	512, 3×3, 1	(512,8,8)
Conv-BatchNorm-Relu (2×)	1024, 3×3, 1	(256,16,16)
Conv-BatchNorm-Relu (2×)	512, 3×3, 1	(128,32,32)
Conv-BatchNorm-Relu (2×)	256, 3×3, 1	(64,64,64)
Conv-BatchNorm-Relu (2×)	128, 3×3, 1	(32,128,128)
Conv-BatchNorm-Relu (2×)	64, 3×3, 1	(6,256,256)
Final Ouptut	−, −, −	(6,256,256)

**Table 3:** Description of the Jigsaw Puzzle Classifier. This classifier is used to pre-train our encoder before fine-tuning for the semantic segmentation task.

Operation Block	Channels, Size of Filter, Padding Value	Size of Output
Input Latent Representation	−, −, −	(512,16,16)
Conv-BatchNorm-Relu (2×)	256, 3×3, 1	(256,16,16)
Conv-BatchNorm-Relu (2×)	128, 3×3, 1	(128,16,16)
Conv-BatchNorm-Relu (2×)	64, 3×3, 1	(64,16,16)
Conv-BatchNorm-Relu (2×)	32, 3×3, 1	(32,16,16)
Conv-BatchNorm-Relu (2×)	16, 3×3, 1	(16,16,16)
Fully Connected-Dropout p=(0.25)	−, −, −	(512)
Fully Connected-Dropout p=(0.25)	−, −, −	(128)
Fully Connected Layer	−, −, −	(256)
Final Ouptut	−, −, −	(16,16)

**Table 4:** Augmentation Parameters used for Data Augmentations in the Albumentation library. The probability of the augmentation applies to its usage during training.

Transformation	Probability	Parameter
ElasticTransform	0.6	alpha = 1, sigma = 25, alpha_affine = 25, border_mode = 0, value = 0
GridDistortion	0.8	num_steps = 5, distort_limit = 0.3, interpolation = 1, border_mode = 4
MotionBlur	0.3	blur_limit = (15, 15)
GaussNoise	0.5	var_limit = (0, 0.0005), mean = 0.001
Random Brightness Contrast	1	brightness_limit = (−0.2, 0.2), contrast_limit = 0.2

**Table 5:** Dice scores [%] of the impact of self-supervised pre-training on semantic segmentation performance on in-house data, LUNA, and IEEE datasets. The best-performing model on each dataset is highlighted in bold. The addition of self-supervised pre-training is particularly helpful in improving the performance on out-of-distribution datasets (LUNA and IEEE).

	In-House	LUNA	IEEE	AVG
Baseline U-Net	83.3%	78.62%	93.21%	81.34%
SSL U-Net	83.3%	88.91%	94.31%	84.12%
SSL + DA U-Net	85.39%	91.87%	95.14%	90.9%

**Table 6:** Dice scores [%] of the impact of attention gates on semantic segmentation performance on in-house data, LUNA, and IEEE datasets. The best-performing model is highlighted in bold.

	In-House	LUNA	IEEE	AVG
Baseline U-Net	83.3%	78.62%	93.21%	81.34%
Attn U-Net	83.54%	83.05%	93.85%	84.31%
SSL Attn U-Net	83.48%	84.96%	94.34%	85.6%
DA Attn U-Net	84.56%	90.91%	94.99%	89.84%
SSL + DA Attn U-Net	85.93%	93.88%	95.88%	92.9%

**Table 7:** Dice scores [%] of the impact of different data augmentations on semantic segmentation performance on in-house data, LUNA, and IEEE datasets. The best-performing model is highlighted in bold.

	In-House	LUNA	IEEE	AVG
Baseline U-Net	83.3%	78.62%	93.21%	81.34%
Patient-DA U-Net	78.56%	88.34%	93.04%	86.85%
Scanner-DA U-Net	79.61%	87.27%	91.62%	86.01%
DA U-Net	80.32%	89.44%	93.21%	87.73%

**Table 8:** Dice scores [%] of the performance of U-Net baseline, an STOA model, and our proposed model using all techniques (SSL, A, DA) on in-house data, LUNA, and IEEE datasets. The best-performing model on each dataset is highlighted in bold.

	In-House	LUNA	IEEE	AVG
Baseline U-Net	83.3%	78.62%	93.21%	81.34%
Johoff-Net	78.91%	94.04%	96.02%	90.3%
(SSL + DA + Attn) U-Net	85.93%	93.88%	95.88%	92.9%

## Data Availability

The public datasets can be accessed from the given references. The in-house dataset can not be made public due to privacy and ethical restrictions, but the associated authors will answer any questions concerning the setup of experiments and data collection as far as possible.

## Abbreviations

The following abbreviations are used in this manuscript:

CT	Computer Tomography
PACS	Picture Archiving and Communication System
